# Expanding the apelin receptor pharmacological toolbox using novel fluorescent ligands

**DOI:** 10.3389/fendo.2023.1139121

**Published:** 2023-03-09

**Authors:** Thomas L. Williams, Robyn G. C. Macrae, Rhoda E. Kuc, Alastair J. H. Brown, Janet J. Maguire, Anthony P. Davenport

**Affiliations:** ^1^ Experimental Medicine & Immunotherapeutics, University of Cambridge, Cambridge, United Kingdom; ^2^ Wellcome-MRC Cambridge Stem Cell Institute, Jeffrey Cheah Biomedical Centre, University of Cambridge, Cambridge, United Kingdom; ^3^ Sosei Heptares, Steinmetz Building, Granta Park, Cambridge, United Kingdom

**Keywords:** GPCR, apelin receptor, apelin, elabela/toddler, fluorescent ligand, stem cells, cardiovascular, kidney

## Abstract

**Introduction:**

The apelin receptor binds two distinct endogenous peptides, apelin and ELA, which act in an autocrine/paracrine manner to regulate the human cardiovascular system. As a class A GPCR, targeting the apelin receptor is an attractive therapeutic strategy. With improvements in imaging techniques, and the stability and brightness of dyes, fluorescent ligands are becoming increasingly useful in studying protein targets. Here, we describe the design and validation of four novel fluorescent ligands; two based on [Pyr1]apelin-13 (apelin488 and apelin647), and two based on ELA-14 (ELA488 and ELA647).

**Methods:**

Fluorescent ligands were pharmacologically assessed using radioligand and functional in vitro assays. Apelin647 was validated in high content imaging and internalisation studies, and in a clinically relevant human embryonic stem cell-derived cardiomyocyte model. Apelin488 and ELA488 were used to visualise apelin receptor binding in human renal tissue.

**Results:**

All four fluorescent ligands retained the ability to bind and activate the apelin receptor and, crucially, triggered receptor internalisation. In high content imaging studies, apelin647 bound specifically to CHO-K1 cells stably expressing apelin receptor, providing proof-of-principle for a platform that could screen novel hits targeting this GPCR. The ligand also bound specifically to endogenous apelin receptor in stem cell-derived cardiomyocytes. Apelin488 and ELA488 bound specifically to apelin receptor, localising to blood vessels and tubules of the renal cortex.

**Discussion:**

Our data indicate that the described novel fluorescent ligands expand the pharmacological toolbox for studying the apelin receptor across multiple platforms to facilitate drug discovery.

## Introduction

G protein-coupled receptors (GPCRs) make up the largest family of signal transducing cell membrane bound receptor proteins that are targeted by clinically approved medicines, with approximately 700 drugs (~35% of all approved drugs) mediating their effects through GPCRs (1–3). Fluorescently labelled ligands are an evolving and increasingly important experimental tool in exploring GPCR pharmacology (4). Major improvements in the brightness and stability of fluorescent dyes, conjugation techniques, and optical equipment and quantification, have meant fluorescent ligands are emerging as safer, easier, and more versatile tools than other classical techniques such as radioligand binding (5–7).

As examples, fluorescently tagged small molecules have been used to successfully probe adenosine A_1_ (8) and A_2A_ receptors (9). These studies also demonstrate that labelled ligands must be carefully validated, as the fluorophore, and linker used to conjugate the fluorophore to the ligand, can have critical effects on pharmacology. In fact, one study showed that conjugation of a fluorophore *via* a dipeptide linker to a non-selective adenosine receptor ligand converted the compound into a high affinity probe exhibiting high selectivity for the adenosine A_3_ receptor (10). On the other hand, cannabinoid CB_2_ receptor (11) and chemokine CXCR4 receptor (12) fluorescent ligands have been shown to retain similar affinities and selectivities compared to their unlabelled analogues, suggesting that the inclusion of fluorophores does not always necessarily alter the pharmacology of the probe.

The apelin receptor, a class A GPCR, was identified in 1993, and paired with its first cognate peptide ligand, apelin, five years later (13, 14). [Pyr^1^]apelin-13 is the most abundant apelin isoform detected in human heart and plasma (14–21) and is secreted from endothelial cells to act in an autocrine/paracrine manner to induce vasodilatation *via* endothelium-dependent mechanisms (20, 21). Uniquely, the apelin receptor is the target of a second endogenous peptide ligand, elabela/Toddler (ELA), translated from the *APELA* gene identified in a conserved region of the genome previously designated as non-coding (22, 23). Biologically active isoforms include ELA-32, ELA-22, ELA-21, and ELA-11, and these share little sequence similarity (~25%) with apelin (22–25). In the adult cardiovascular system, the apelin receptor mediates venous and arterial vasodilatation (20, 26–30) and positive inotropic actions leading to increased cardiac contractility and cardiac output in animal models (31–33) and humans (28, 29), through activation of apelin receptors on cardiomyocytes (34). ELA plays a critical role in embryogenesis, where the peptide acts as the primary activator of the apelin receptor to drive cardiac development (22, 23). ELA peptide is also present in the adult cardiovascular system, in the endothelium and plasma (at higher levels than apelin), but not in adult cardiomyocytes (35). However, exogenous ELA administration still increases cardiac contractility and output through action on the apelin receptor *in vivo*. The apelin receptor, therefore, presents a highly tractable drug target for cardiovascular disorders characterised by high blood pressure and a failing heart, such as pulmonary arterial hypertension (PAH) (36–41).

Apelin-13 peptide conjugated to lissamine rhodamine dye has been used previously to image small-scale internalisation of apelin receptor (42, 43). Subsequently, a far-red fluorescent peptide with sub-nanomolar affinity for the apelin receptor has also been used to visualise internalisation (44). It is clear that apelin peptides are tractable to fluorescent labelling, but the full range of advantages that fluorescent ligands offer has not been fully exploited for this GPCR target. Here, we outline the design of four directly labelled, novel fluorescent peptide ligands targeting the apelin receptor. Two fluorescent apelin ligands were based on a modified apelin-13 peptide scaffold that shows high affinity for the apelin receptor (45, 46), and which has been used previously for the well characterised [^125^I]-apelin-13 radioligand. Two fluorescent ELA ligands were also synthesised, based on the endogenous ELA-14 peptide – the first such ligands to be designed to our knowledge. All four ligands were validated in binding and functional *in vitro* assays, and were shown to retain binding affinity and behave as agonists at their target receptor. Far-red apelin647 fluorescent ligand was used to explore apelin receptor visualisation, binding, and internalisation in an artificially expressing CHO-K1 cell line, in conjunction with a high content screening system. Fluorescent ligands were subsequently used to visualise apelin receptor binding in a clinically relevant human embryonic stem cell-derived cardiomyocytes (hESC-CM) model, and in human kidney sections, demonstrating suitability as a replacement for antibodies in immunohisto-/cyto-chemistry assays. The findings establish our novel fluorescent apelin receptor ligands as safe, easy-to-use, and versatile tools that offer many more measurable parameters to improve upon classical techniques such as radioligand binding. Additionally, novel fluorescent ELA peptides may provide further insight into whether the second endogenous ligand exhibits a different binding and pharmacological profile to apelin.

## Materials and methods

### Synthesis of fluorescent ligands

Apelin488, apelin647, ELA488, and ELA647 were synthesised *via* Fmoc solid-phase synthesis to custom orders by Cambridge Research Biochemicals (CRB; Cleveland, UK). Sequences are provided in the Results section and the described fluorescent ligands are commercially available as custom syntheses.

### CHO-K1 cell culture

CHO-K1 cells were maintained in 20-25 mL DMEM/F-12 (1:1) nutrient mix + _L_-glutamine (ThermoFisher Scientific), supplemented with 10% FBS (ThermoFisher Scientific) in the presence of 0.1 mg/mL Normocin antibiotic formulation (InvivoGen) in T175 cell culture flasks (Corning). Cells were grown to confluency before washing with sterile phosphate buffered saline (PBS, ThermoFisher Scientific), and subsequent passaging with 3 mL porcine trypsin for 3 mins. Trypsin dissociation was neutralised with 7 mL culture media, before cell suspensions were spun at 300 xg for 5 mins. Media was aspirated before cell pellets were resuspended in 10 mL media for replating in flasks or experimental plates. For determination of cell numbers, 3 mL of the cell suspension was diluted 1:10 in media, and 10 μL of the resulting suspension counted using a haemocytometer. CHO-K1 cells stably expressing recombinant apelin receptor (referred to as CHO-APLNR) were a kind gift from Mr Jason Brown at Sosei Heptares, and were cultured in the same manner as wild-type CHO-K1 cells.

### hESC/hESC-CM cell culture & differentiation

Culturing and differentiation was performed using a protocol adapted from Cheung et al., 2014 (47). In brief, undifferentiated H9 human embryonic stem cells (hESCs) were maintained in CDM-BSA media, comprised of Iscove’s Modified Dulbecco’s Medium (IMDM)/F-12 (1:1) nutrient mix, supplemented with 15 μg/mL transferrin (R&D Systems), 7 μg/mL insulin, 450 mM monothioglycerol, 1% chemically defined concentrated lipids (ThermoFisher Scientific), 5 mg/mL bovine serum albumin (Europa Bio Products), 100 U/mL penicillin-streptomycin (ThermoFisher Scientific), 12 ng/mL fibroblast growth factor 2 (FGF2, Qkine Ltd), and 10 ng/mL activin-A (Qkine Ltd). Cells were grown in 6-well plates (Corning) coated with 0.1% gelatin in sterile PBS. Media was changed every day. Cells were grown to confluency before washing with sterile PBS, and subsequent passaging with collagenase IV (ThermoFisher Scientific) for 3 mins. Collagenase dissociation was neutralised with media, before cells were scraped and triturated for collection into tubes. Cells were allowed to settle at the bottom of the tubes before aspiration of the media and subsequent resuspension of cells in fresh media for replating.

Dissociated H9 hESCs were collected as a triturated suspension. A 10 μL volume of cell suspension was mixed 1:1 with 0.4% trypan blue solution (ThermoFisher Scientific) for counting in a Countess Automated Cell Counter (Invitrogen). Remaining cells were spun in at 300 xg for 3 mins, before resuspending in CDM-BSA media, supplemented with 12 ng/mL FGF2, 30 ng/mL activin-A, and 10 μM Rho-associated protein kinase inhibitor (ROCKi, Insight Biotech) to give a final cell concentration of 80 k/well in 6-well plates coated with Matrigel (Corning). After incubation for 4 h, mesoderm induction was performed by aspirating media and replacing with 2 mL/well CDM-BSA media, supplemented with 20 ng/mL FGF2, 50 ng/mL activin-A, 10 μM phosphoinositide 3-kinase inhibitor (Ly294002, Stratech), and 10 ng/mL bone morphogenetic protein 4 (BMP4, R&D Systems). After a further incubation for 42 h, media was aspirated, and cells washed with sterile PBS. Cells were covered with CDM-BSA media, supplemented with 8 ng/mL FGF2, 10 ng/mL BMP4, 1 μM retinoic acid, and 1 ng/mL Wnt signalling pathway inhibitor (IWR1-endo, Tocris). Cells were re-fed with this media every 48 h, for 4 days, before swapping to CDM-BSA media, supplemented with 8 ng/mL FGF2 and 10 ng/mL BMP4. Cells were refreshed with media every other day, until spontaneous beating of differentiated cardiomyocytes (hESC-CMs) was observed.

After ≥ 14 days, media was aspirated, and cells washed with sterile PBS. Cells were subsequently dissociated with TrypLE Express (ThermoFisher Scientific) for 10 mins, before neutralising with CDM-BSA media, supplemented with 5 μg/mL DNase I (New England BioLabs) to prevent cell clumping. Cells were collected into tubes and spun at 300 xg for 3 minutes. Media was aspirated off cell pellets before resuspension in CDM-BSA, supplemented with 10 μM ROCKi and plating in 6-well plates coated with Matrigel. Cells were incubated overnight. Next, media was removed and replaced with a lactate selection media, comprised of DMEM no glucose, no pyruvate (ThermoFisher Scientific), supplemented with 1X MEM non-essential amino acids (ThermoFisher Scientific), and 4 mM sodium L-lactate solution in HEPES buffer.

### Generation of shRNA apelin receptor knockdown hESC-CMs

The inducible apelin receptor knockdown used in this study has been used previously by our group. For validation, experimental findings, and relevant methods, see Macrae et al., 2022 (48). In brief, a tetracycline inducible, short hairpin RNA apelin receptor knockdown system was designed using the single-step optimised inducible knockdown system (sOPTiKD) (49). These authors outline a pAAV-Puro_siKD vector targeting the AAVS1 locus for transgene expression, and carrying the short hairpin RNA and tetracycline response expression cassettes, and a puromycin resistance gene. The pAAV-Puro_siKD vector was a kind gift from Professor Ludovic Vallier (Addgene plasmid #86695; http://n2t.net/addgene:86695; RRID: Addgene_86695).

H9 hESCs, cultured in 6-well plates coated with 0.1% gelatin, were aspirated of media, and washed with sterile PBS, before incubation with 1 mL Opti-MEM reduced serum media. Two transfection mixtures were made. Mixture A was comprised of 10 μL Lipofectamine 2000 Transfection Reagent (Thermofisher Scientific) in 240 uL Opti-MEM per well to be transfected. Mixture A was incubated for 5 mins. Mixture B was comprised of 2 μg shRNA vector and 2 μg of 2 AAVS1 zinc finger nuclease plasmids (also described in Bertero et al., 2016) in 250 μL Opti-MEM per well to be transfected. Mixture A and Mixture B were added 1:1 and incubated for 20 mins at room temperature to allow complexes to form. Subsequently, 500 μL of the complex solution was added to each well to be transfected. Cells were incubated for 24 h at 37°C, 5% CO_2_, humidified. Cells were then aspirated and washed with sterile PBS and maintained in CDM-BSA media, refreshed daily, until ~80% confluency was observed. Cells were swapped to CDM-BSA media containing 1 μg/mL puromycin, refreshed daily, to select for colonies that had successfully taken up the construct. Colonies were manually isolated using a pipette, and expanded in colonies before undergoing the cardiomyocyte differentiation protocol outlined above.

### Cell membrane preparation and tissue homogenisation

CHO-APLNR cell membrane preparations were made by dissociating cells grown to confluency in T175 flasks using 3 mL trypsin or TrypLE Express for 3 mins. Dissociating reagent was neutralised with 7 mL cell media. Cell suspensions were transferred to tubes and spun at 1000 xg for 10 mins at 4°C. Pellets were resuspended in ice-cold TRIS wash buffer (50 mM TRIS-HCl in deionised water, pH balanced to 7.4 at room temperature, as used during wash steps of subsequent radioligand binding experiments), and triturated, to induce complete hypotonic lysis of cells. Cells were spun a second time at 21,000 xg for 20 mins at 4°C, to obtain a crude membrane fraction. Supernatant was discarded and membrane preparations resuspended in TRIS wash buffer. Cell membrane preparations were stored at -70°C until use in experiments.

Surgical samples of human left ventricle (HLV) tissue were obtained with informed consent and ethical approval (05/Q104/142). Tissues were snap frozen in liquid nitrogen before storage at -80°C. On the day of preparation, tissue was chopped finely with a razor blade on ice, before subsequent homogenisation using a Polytron Homogenizer (Thomas Scientific), in homogenisation buffer (50 mM Tris-HCl, 5 mM MgCl2, 5 mM EDTA, 1 mM EGTA, 1:500 protease inhibitor cocktail containing aprotinin and amastatin, balanced at pH 7.4) at 4°C. The resulting homogenate was subsequently spun at 1000 xg for 2 mins at 4°C. Supernatant was transferred to another set of tubes (pellet discarded) for a high speed spin at 40,000 xg, for 30 mins at 4°C. Supernatant was then discarded, and the remaining pellet resuspended in 2.5 mL/g of homogenisation buffer, before a second high speed spin at 40,000 xg, for 30 mins at 4°C. The supernatant was discarded, and the remaining pellet was resuspended in HEPES buffer (50 mM HEPES, balanced at pH 7.4 at room temperature) and stored in the freezer at -70°C until use in assays.

### Protein assay

Protein concentration of protein preparations (cells or homogenised tissue) was determined using a DC Protein Assay (Bio-Rad), in accordance with the manufacturer’s instructions. In brief, the solubilising buffer provided in the kit was warmed on top of a water bath heated to 80°C to prevent precipitation of buffer components. Frozen protein samples were thawed and mixed 1:1 (typically 100 μL) with the solubilising buffer. The mix was heated in the 80°C water bath for 30 mins. Samples were then spun at 11,000 xg for 5 mins. 50 μL of supernatant was serially diluted with 50 μL solubilising buffer down to 1:128. A bovine serum albumin (BSA) standard at 1.5 mg/mL in solubilising buffer was prepared and diluted down to 0.1875 mg/mL to produce a standard curve in the assay. After adding 5 μL blank controls (solubilising buffer), BSA standard, or sample supernatant dilutions to a clear-bottomed 96 well plate, kit reagents A’ (alkaline copper tartrate solution) and B (dilute folin reagent) were added at 25 and 200 μL respectively. After incubating for 5 mins at room temperature, absorbance at 450 nm was measured using a FLUOstar Omega microplate reader (BMG LABTECH). The BSA standard curve was plotted, and concentrations of the protein samples interpolated.

### Radioligand binding

Radioligand binding was performed using [Glp^65^,Nle^75^,Tyr^77^][^125^I]-apelin-13 (referred to as [^125^I]-apelin-13 throughout; PerkinElmer), using protocols adapted from Katugampola et al., 2001 (50); Read et al., 2016 (51); and Yang et al., 2017 (35). The radiolabel has a specific activity of 2200 Ci/mmol. Binding buffer was comprised of 50 mM TRIS-HCl and 5 mM MgCl_2_, balanced to pH 7.4 at room temperature. Wash buffer was the same but with no MgCl_2_. Protein samples were diluted in binding buffer to give final assay concentrations of 1 mg/mL or 1.5 mg/mL for cell preparations or homogenised tissue respectively. Plastic-ware used in radioligand binding experiments was coated with Sigmacote, a siliconising reagent that forms a covalent film that reduces sticking of the radiolabel.

Competition binding was performed to assess ability of fluorescent ligands to compete with [^125^I]-apelin-13 to the apelin receptor. Protein sample was mixed 1:1:1 with 0.1 nM [^125^I]-apelin-13, and a concentration range (1 pM – 1 μM) of competing ligand, diluted in binding buffer. Total binding was determined in the absence of competing fluorescent ligand. Non-specific binding was determined in the presence of 5 μM [Pyr^1^]apelin-13. At the end-point, samples were spun at 20,000 xg for 10 mins at 4°C to terminate equilibrium. Supernatant was aspirated, and pellets were resuspended and triturated in 500 μL ice-cold wash buffer all over ice. Samples were spun a second time at 20,000 xg for 10 mins at 4°C, before aspiration of the supernatant. Radioactivity in pellets was counted using a Cobra II model 5003 gamma counter (Packard). Competition binding data were analysed using the nonlinear one site Fit K_i_ [2] model using GraphPad Prism version 6.07, with constraints for the radiolabel concentration (0.1 nM) and K_D_ (0.076 nM, previously determined by saturation analysis in homogenised human left ventricle) input. The software was also used to calculate binding affinities (K_i_ values) of competing ligands using the Cheng-Prusoff equation.

### Dynamic mass redistribution assay

Apelin receptor downstream signalling was determined using an *in vitro* BIND technology dynamic mass redistribution assay (SRU Biosystems). CHO-APLNR cells were seeded in 96-well biosensor plates (SRU Biosystems) at a density of 50K/well, maintained in 95 µL feeding media, and incubated for 24 h at 37°C, 5% CO_2_, humidified. The following day, the plate was loaded onto the SRU Biosystems BIND PROFILER TURBO reader, and left to acclimatise to room temperature for 20 mins before a baseline read was taken over 10 mins. To test novel fluorescent compounds for agonist activity, cells were treated with test compounds (5 µL additions in PBS) over a concentration range to generate an 8-point concentration-response curve. The biosensor plate was read for a subsequent 60 mins. Responses, measured in maximal change in peak wavelength value from baseline (ΔPWV), were fitted to four parameter logistic concentration response curves in GraphPad Prism version 6.07 for Windows (GraphPad Software) to determine compound concentrations inducing half maximal response (EC_50_), and *p*D_2_ values (-log10 EC_50_) were calculated.

### Internalisation assay

Internalisation of apelin receptor following ligand treatment was determined using an *in vitro* PathHunter GPCR Internalisation Assay (Eurofins). The manufacturer’s protocol was modified in accordance with previous in-house optimisation. All reagents were provided in the assay kit. U2OS cells overexpressing human apelin receptor (AGTRL1) were seeded in cell plating reagent into 96-well plates and incubated for 48 h at 37°C, 5% CO_2_, humidified. To test novel fluorescent compounds for agonist activity, wells were treated with test compounds over a concentration range in Cell Plating Reagent to generate an 8-point concentration-response curve. Cells were then incubated for 90 mins at 37°C, 5% CO_2_, humidified before treatment with Working Detection Solution (Cell Assay Buffer, Substrate Reagent 1, Substrate Reagent 2), and incubation for 2 h at room temperature, protected from light. Plates were read in a chemiluminescence detecting LumiLITE Microplate Reader (DiscoveRx, Fremont, CA, USA). Responses, measured in Relative Light Units (RLU), were fitted to four parameter logistic concentration response curves in GraphPad Prism version 6.07 for Windows (GraphPad Software) to determine compound concentrations inducing half maximal response (EC_50_), and pD_2_ values (-log10 EC_50_) were calculated.

### Fluorescent ligand binding in CHO-APLNR cells

Cells were seeded in CellCarrier-96 Ultra Plates at a density of ~10 k/well. After a wash with HBSS, CHO-APLNR cells were treated with apelin647. For time-course experiments, CHO-APLNR were treated with 300 nM of the fluorescent ligand for 0 – 90 mins, in the dark at room temperature. For saturation binding experiments, CHO-APLNR were treated with a concentration range of 1 – 300 nM of the fluorescent ligand for 90 mins, in the dark at room temperature. Non-specific binding was determined in the presence of 10 µM [Pyr^1^]apelin-13. Untransfected CHO-K1 cells were also used as controls. For competition binding experiments, concentration ranges of [Pyr^1^]apelin-13 and ELA-14 (0.1 nM – 10 µM), or MM54, F13A, and CMF-019 (0.1 nM – 30 µM) were used to compete against a 30 nM concentration of apelin647. At the end-points, CHO-APLNR were washed with HBSS before fixation with 4% formaldehyde. CHO-APLNR were washed with HBSS before treatment with Hoechst 33342 nuclear marker prepared at 10 µg/mL in HBSS for 15 mins. Competition binding data were analysed using the nonlinear one site Fit Ki [2] model using GraphPad Prism version 6.07, with constraints for the fluorescent ligand concentration (30 nM) and K_D_ (78.94 nM, previously determined by fluorescent saturation analysis described above) input. The software was also used to calculate binding affinities (K_i_ values) of competing ligands using the Cheng-Prusoff equation. For internalisation experiments, CHO-APLNR cells were treated with WGA-555 (10 µg/mL) and Hoechst 33342 nuclear marker (10 µg/mL) in HBSS for 15 mins, before a wash and subsequent treatment with 300 nM red fluorescent apelin ligand for 2 mins. After a final wash with HBSS, cells were imaged every 30 seconds at 37 °C and 5% CO_2_ over a 90 mins time-course using an Opera Phenix High Content Screening System (PerkinElmer, see below).

### Fluorescent ligand binding in hESC-CMs

Cells were seeded in CellCarrier-96 Ultra Plates (PerkinElmer) at a density of ~40 k/well. After a wash with HBSS, wild-type or apelin receptor knockdown hESC-CMs were treated with 300 nM red fluorescent apelin, made up in HBSS, for 90 mins in the dark at room temperature. Non-specific binding was determined in the presence of 10 µM [Pyr^1^]apelin-13. hESC-CMs were then washed with HBSS before fixation with 4% formaldehyde for 3 mins. hESC-CMs were washed with HBSS before treatment with Hoechst 33342 nuclear marker prepared at 10 µg/mL in HBSS for 15 mins. After a final HBSS wash, hESC-CMs were maintained in 100 µL HBSS for imaging using an Opera Phenix High Content Screening System (PerkinElmer, see below).

### Opera phenix high content screening

For imaging studies, cells in CellCarrier-96 Ultra Plates (PerkinElmer) were imaged using a 40x/NA1.1 water immersion objective. The device was set to measure an image field within each well, where the field is made up of a 4 x 4 or 5 x 5 grid of imaging regions (where each region is 250 x 250 μm in area). After using Z stacking to find an appropriate focal plane, cells were visualised and imaged at a focal depth of 0.5 μm above the bottom of the plate in all experiments. A profile with four fluorescent channels was used; a first channel (blue), with excitation of 405 nm and emission filter of 435-480 nm for Hoechst 33342 nuclear marker; a second channel (green), with excitation of 488 nm and emission filter of 500-550 nm for Alexa Fluor 488 or eGFP; a third channel (gold), with excitation of 561 nm and emission filter of 570-630 nm for Alexa Fluor 555; and a fourth channel (red), with excitation of 631 nm for Alexa Fluor 647. In all instances, excitation laser intensity was set at 50% of the Opera Phenix maximum, with a 50 ms exposure time. In all experiments, cells were also imaged using digital phase contrast, an optical method that translates phase shifts in light passing through cell matter in the plates to changes in brightness. Digital phase contrast images were used alongside fluorescent nuclear and membrane markers to identify and segment cells.

Following image acquisition, several cell parameters were quantified using in-built Harmony High Content Imaging and Analysis Software (PerkinElmer). For analysis, images were filtered, and the contrast of the Hoechst nuclear blue channel was increased five-fold to ensure cells were distinguished from the background. WGA-555 positivity and digital phase contrast was used to find the membrane and outer limits of individual cells. A membrane collar of ± 0.2 µm either side of the outer limit of cells was established as a surrogate for the cell membrane compartment. Intracellular fluorescence was calculated as fluorescence identified in the membrane collar subtracted from whole cell fluorescence. Mean fluorescence intensities were calculated for membrane and whole cell compartments in observed cells in the selected population, given as fluorescence units provided by Harmony software.

### Fluorescent ligand binding in human kidney tissue

Frozen human kidney tissue sections (10 µm), were cut using a cryostat onto microscopy slides. On the day of experimentation, slides were allowed to acclimatise to room temperature in a humid environment before being washed with Hank’s balanced salt solution (HBSS; Merck) and subsequently treated with 1 µM apelin488 or 1 µM ELA488, made up in HBSS, for 30 mins in the dark at room temperature. Non-specific binding was determined in the presence of 10 µM [Pyr^1^]apelin-13 or ELA-14 for apelin488 and ELA488 respectively. Slides were washed with HBSS before fixation with 4% formaldehyde for 3 mins. Note that apelin488 and ELA488 were used in these experiments as a damaged filter set meant that emitted light from fluorophores in the far-red spectrum could not be appropriately visualised by the imaging platform (see below).

Immunohistochemistry was then performed on fixed tissue sections to stain endothelial cells. Non-specific staining was blocked with PBS containing 10% donkey sera in PBS for 2 h at room temperature. Tissue sections were incubated overnight at 4 °C with a primary monoclonal (F8/86) mouse antibody raised against human von Willebrand factor (M0616; Dako; 1:50 dilution), prepared in PBS with 1% donkey sera, 0.1% Tween-20, and 3.3 mg/ml bovine serum albumin. Buffer-treated controls were incubated with PBS with 1% donkey sera, 0.1% Tween-20, and 3.3 mg/ml bovine serum albumin alone. After 24 h, slides were washed 3x with 100 μL PBS with 0.1% Tween-20 before incubation with the secondary polyclonal Donkey Anti-Mouse IgG H&L antibody conjugated to Alexa Fluor 555 (ab150106; Abcam; 1:200) prepared at 0.01 mg/ml in PBS with 1% donkey sera, 0.1% Tween-20, and 3.3 mg/ml bovine serum albumin, for 1 h at room temperature. Slides were washed 3x with PBS before incubation with Hoechst 33342 nuclear stain (H3570; Invitrogen) prepared at 10 μg/ml in PBS for 15 mins at room temperature in the dark. After a final 3x washes with PBS, slides were blotted dry with lint-free tissue, mounted with ProLong Gold Antifade Mountant, covered with a cover slip, and left at room temperature in the dark to set (≥ 48 h).

Automated fluorescent images (16 bit, 0.325 x 0.325 μm scaling per pixel) of fixed and mounted human tissue stained with fluorescent markers were acquired using an Axio Scan Z1 slide scanner (Zeiss) slide scanner with a Plan-Apochromat 20x/NA0.8 M27 objective lens connected to a Hamamatsu Orca Flash camera. An initial brightfield scan was performed to visualise tissue on slides, and a spline contour tool was used to outline the tissue to minimise the total region imaged. A profile with three fluorescent channels was used; a first channel (blue), with an LED-Module 385 nm light source set at 10% intensity and 10 ms exposure time at a depth of focus of 1.45 μm for Hoechst 33342 nuclear marker (405 nm wavelength); a second channel (green), with an LED-Module 475 nm light source set at 80% intensity and 30 ms exposure time at a depth of focus of 1.64 μm for 488 nm wavelengths; and a third channel (yellow/orange), an LED-Module 567 nm light source set at 80% intensity and 30 ms exposure time at a depth of focus of 1.88 μm for 555 nm wavelengths. Focal depths were determined by the slide scanner’s in-built Z stacking autofocusing. All acquired images were saved and visualised using ZEN software (Zeiss) and/or Orbit Image Analysis (ORBIT) software. Regions of interest were identified using the spline contour, and mean fluorescent signal (in grayscales) was provided by ZEN software (Zeiss).

### Data analysis and statistics

Quantitative data are expressed as mean ± standard error of the mean (s.e.m.). Graphical presentation and statistical tests were performed using GraphPad Prism version 6.07 for Windows (GraphPad Software). For radioligand competition binding experiments, K_i_ values were determined using Cheng-Prusoff methodology. For *in vitro* GPCR assays, EC_50_, *p*D_2_, and E_max_ values were calculated using GraphPad Prism version 6.07 for Windows. Statistical tests and n numbers are indicated in figure legends where used. A p value of < 0.05 was determined as significant.

## Results

**Figure 1:**
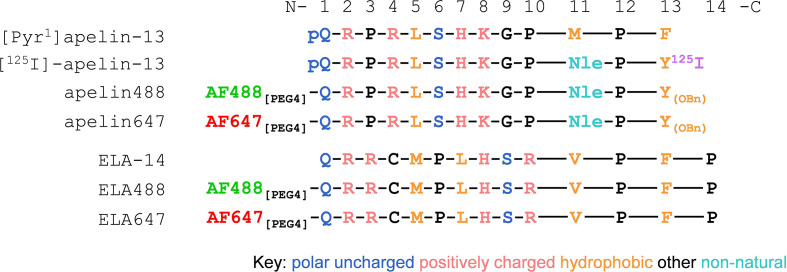
Schematic showing 13-/14-mer single letter amino acid sequences for apelin and ELA based peptide ligands respectively. Sequences read from the N-termini (left) to C-termini (right). For apelin tool ligands, methionine (M) was replaced with the non-natural amino acid norleucine (Nle) at position 11, and the C-terminal phenylalanine (F) was replaced with tyrosine (Y) at position 13. pQ denotes the natural pyroglutamate residue found in the endogenous [Pyr^1^]apelin-13 peptide. ^125^I denotes the iodine^125^ present at the C-terminus in the [^125^I]-apelin-13 radiolabel. OBn denotes O-benzene. Fluorescent dyes structurally identical to Alexa Fluor 488 and 647 (AF488 and AF647) were conjugated to the N-terminal residues of peptides *via* a 4-mer polyethylene glycol (PEG4) linker to generate the fluorescent ligands used in this study.

The schematic in **Figure 1** shows the amino acid sequences of the endogenous apelin receptor peptide ligands, [Pyr^1^]apelin-13 and ELA-14, and the modifications incorporated in the various tool ligands used in this study. Fluorescent apelin peptides conjugated to dyes structurally identical to Alexa Fluor 488 and 647, are referred to as apelin488 and apelin647, whilst ELA peptides conjugated to these dyes are referred to as ELA488 and ELA647.

### Fluorescent ligands bind, and function as agonists, at the apelin receptor

**Figure 2:**
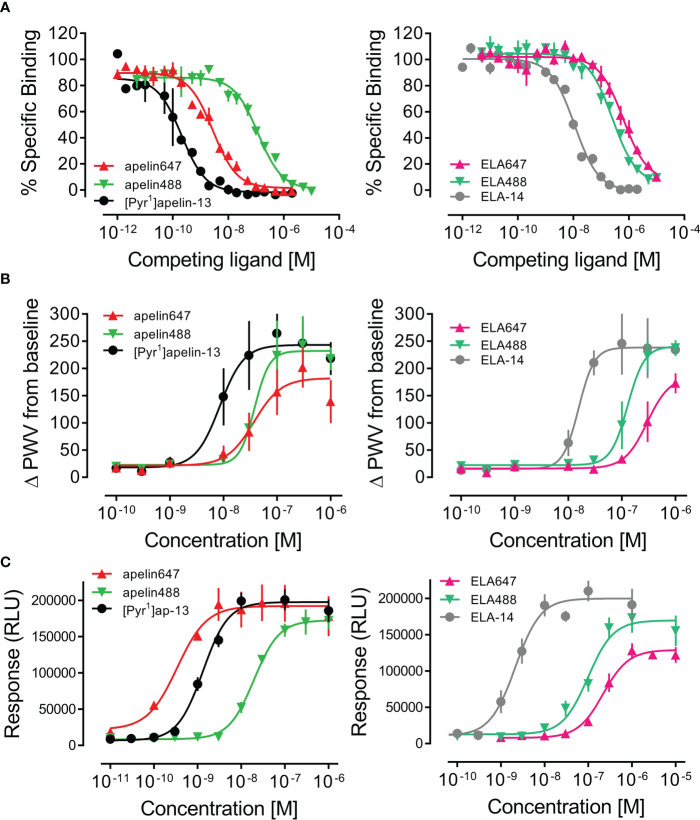
*In vitro* pharmacological validation of fluorescent apelin receptor ligands. **(A)** Competition binding curves show specific binding of apelin ligands (left) and ELA ligands (right) against a 0.1 nM concentration of [^125^I]-apelin-13 in human left ventricle tissue homogenate (1.5 mg/mL protein). **(B)** Concentration-response curves for apelin ligands (left) and ELA ligands (right) in an *in vitro* dynamic mass redistribution assay, showing response as change in peak wavelength value (ΔPWV) from baseline. Data are expressed as mean ± s.e.m., n = 3. **(C)** Concentration-response curves for apelin ligands (left) and ELA ligands (right) in an *in vitro* internalisation assay, showing response in relative light units (RLU). Data are expressed as mean ± s.e.m., n = 3. In all panels, left column shows [Pyr^1^]apelin-13 = ●, apelin488 = ▼, apelin647 = ▲, and right column shows ELA-14 = ●, ELA488 = ▼, ELA647 = ▲.

The well characterised [^125^I]-apelin-13 radiolabel was used to validate binding of apelin and ELA fluorescent ligands in human heart homogenate tissue (**Figure 2A**). [Pyr^1^]apelin-13 bound with high, sub-nanomolar affinity (*p*K_i_ value of 10.05 ± 0.11), whilst apelin647 binding affinity was approximately 13-fold lower (*p*K_i_ value of 8.92 ± 0.05), and apelin488 binding affinity was approximately 575-fold lower (*p*K_i_ value of 7.29 ± 0.07). ELA-14 bound with nanomolar affinity (*p*K_i_ value of 8.33 ± 0.05). Again, fluorescent ligands bound with lower affinity than the endogenous parent ligand; ~23-fold lower for ELA488 (*p*K_i_ value of 6.96 ± 0.07), and ~58-fold lower for ELA647 (*p*K_i_ value of 6.57 ± 0.08). Nevertheless, all novel fluorescent ligands were able to compete for binding with [^125^I]-apelin-13, indicating specific engagement with the apelin receptor in human heart tissue.

Fluorescent ligands were further validated for pharmacological activity at the apelin receptor using *in vitro* assays. In a dynamic mass redistribution assay (**Figure 2B**), [Pyr^1^]apelin-13 displayed a *p*D_2_ value of 8.07 ± 0.20 and an E_max_ value of 243.09 ± 26.03 ΔPWV. Comparable with binding, apelin488 and apelin647 were less potent in the dynamic mass redistribution assay, displaying *p*D_2_ values of 7.42 ± 0.15 and 7.43 ± 0.18, and E_max_ values of 231.98 ± 24.11 and 182.01 ± 24.56 ΔPWV, respectively. ELA-14 was less potent than [Pyr^1^]apelin-13, displaying a *p*D_2_ value of 7.81 ± 0.11 (E_max_ = 238.27 ± 18.00 ΔPWV). Again, the inclusion of fluorophores appeared to reduce the potencies of ELA488 and ELA647, with *p*D_2_ values of 6.90 ± 0.10 (E_max_ = 240.32 ± 27.91 ΔPWV) and 6.53 ± 0.13 (E_max_ = 185.23 ± 36.82 ΔPWV) respectively.

Finally, fluorescent ligands were assessed in an internalisation assay (**Figure 2C**). Here, [Pyr^1^]apelin-13 induced internalisation with a *p*D_2_ value of 8.88 ± 0.07 and E_max_ of 197731 ± 6502 RLU. Apelin488 was ~15-fold less potent, displaying a *p*D_2_ value of 7.71 ± 0.07 (E_max_ of 172593 ± 6453 RLU), whilst apelin647 displayed a *p*D_2_ value of 9.47 ± 0.37 (E_max_ of 192282 ± 11049 RLU). ELA-14 was essentially equipotent with [Pyr^1^]apelin-13, with a *p*D_2_ value of 8.67 ± 0.09 and an E_max_ of 199938 ± 8256 RLU. Both ELA488 and ELA647 were less potent than the unlabelled ELA-14 parent compound, displaying *p*D_2_ values of 7.01 ± 0.08 and 6.66 ± 0.08 respectively. Whilst ELA488 induced full internalisation at the top concentration (E_max_ of 169693 ± 9461 RLU), ELA647 appeared to induce less overall internalisation of apelin receptors in this assay (E_max_ of 128992 ± 6218 RLU).

### Apelin647 can be used as a robust tool in conjunction with high content imaging to assess apelin receptor pharmacology

**Figure 3:**
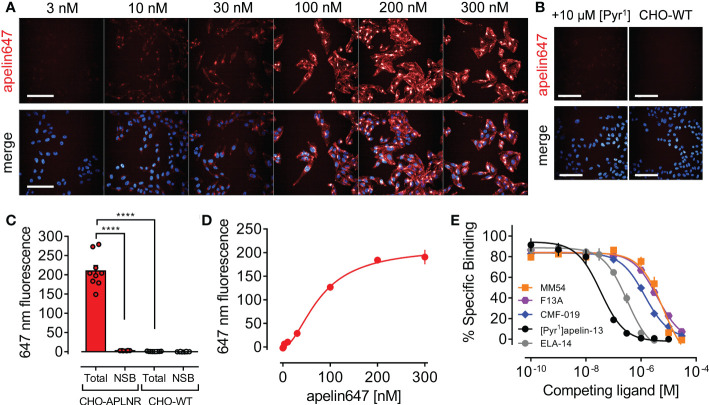
Validation of apelin647 as a robust tool for qualitatively and quantitatively assessing apelin receptor pharmacology using high content imaging. **(A)** Representative confocal fluorescent images of CHO-APLNR cells treated with a concentration range up to 300 nM of apelin647 (visualised in red). Bottom row shows a merge with Hoechst 33342 nuclear marker (visualised in blue). n = 3 independent experiments. Scale bars indicate 100 µm. **(B)** Control images show CHO-APLNR treated with 300 nM apelin647 in the presence of a saturating 10 µM concentration of unlabelled [Pyr^1^]apelin-13 (left column), or wild-type CHO-WT cells treated with 300 nM apelin647 (right column). n = 3 independent experiments. Scale bars indicate 100 µm. **(C)** Bar chart shows quantified total and non-specific (NSB) binding of 300 nM apelin647 in CHO-APLNR and CHO-WT, where non-specific binding was determined in the presence of 10 µM [Pyr^1^]apelin-13. Statistical significance was determined using a one-way ANOVA, with Tukey’s correction for multiple comparisons looking for differences between treatment groups. ****p < 0.0001. Data are expressed as mean ± s.e.m., n = 3 independent experiments. **(D)** Quantified specific saturation binding curve for apelin647 (1-300 nM) in CHO-APLNR. Data are expressed as mean ± s.e.m., n = 3. **(E)** Competition binding curves for [Pyr^1^]apelin-13 (●, n = 4), ELA-14 (●, n = 3), MM54 (■, n = 3), F13A (●, n = 1), and CMF-019 (♦, n = 1) against a 30 nM concentration of apelin647 in CHO-APLNR.

Given the potential for use of fluorescent GPCR ligands in studying receptor pharmacology, high content imaging was used to qualitatively and quantitatively assess apelin647 binding in CHO-APLNR cells (**Figure 3**). Apelin647 fluorescence bound in a concentration-dependent manner (**Figure 3A**), and binding was confirmed as specific due to near total loss of fluorescent signal in CHO-APLNR co-incubated with a saturating 10 μM concentration of unlabelled [Pyr^1^]apelin-13, and in wild-type CHO-K1 cells that do not endogenously express the apelin receptor (**Figure 3B**). Quantification of fluorescent signal (**Figure 3C**) confirmed that total binding of 300 nM apelin647 (209.79 ± 42.50 mean fluorescence) in CHO-APLNR was significantly reduced by co-incubation with [Pyr^1^]apelin-13 (2.78 ± 0.38 mean fluorescence; p < 0.0001). Additionally, total binding was significantly higher than that observed in wild-type CHO-K1 cells treated with 300 nM apelin647 (0.70 ± 0.45 mean fluorescence; p < 0.0001). The data suggest that binding of apelin647 is highly specific, with almost no fluorescent signal detected in controls.

Quantification of the concentration-dependent fluorescent signal of apelin647 in CHO-APLNR (**Figure 3D**), demonstrated that binding was saturable at the highest concentration tested (300 nM), displaying a K_D_ value of 78.94 ± 7.02 nM. A 30 nM concentration was used to assess competition for apelin receptor binding with several other ligands (**Figure 3E**). [Pyr^1^]apelin-13 competed for binding with a *p*K_i_ value of 7.51 ± 0.07, whilst ELA-14 competed with a *p*K_i_ value of 6.55 ± 0.08. Reported apelin receptor peptide antagonists, MM54 and F13A, had *p*K_i_ values of 5.24 ± 0.09, and 5.46 respectively. CMF-019, a G protein-biased small molecule apelin receptor agonist displayed a *p*K_i_ value of 5.91.

### Apelin647 induces internalisation of the apelin receptor that can be visualised and quantified

**Figure 4:**
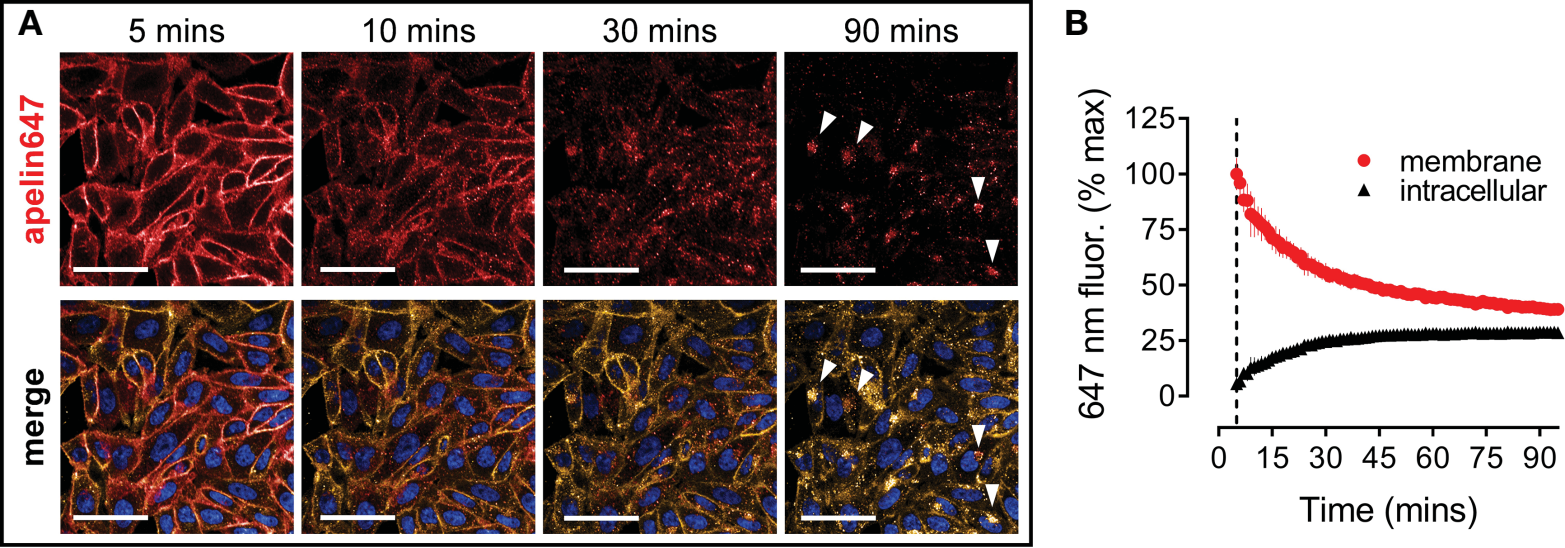
Apelin647 induces apelin receptor internalisation that can be visualised using high content imaging. **(A)** Representative confocal fluorescent images of CHO-APLNR cells treated with 300 nM apelin647 over a 90 minute time-course (imaging commenced at 5 mins). Bottom row shows a merge with wheat-germ agglutinin-555 membrane marker (visualised in gold) and Hoechst 33342 nuclear marker (visualised in blue). White arrows indicate perinuclear staining. Scale bars indicate 50 µm. n = 3 independent experiments. **(B)** Quantified membrane (●) and intracellular (▲) localisation of apelin647 fluorescence (expressed as % max membrane fluorescence observed at time 5 mins) over the 90 minute time-course. Data are expressed as mean ± s.e.m., pooled from n = 3 independent experiments.

Following the data showing apelin647 potently induces apelin receptor internalisation (**Figure 2C**), this phenomenon was visualised and quantified using high content imaging (**Figure 4**). CHO-APLNR cells were treated with 300 nM apelin647 for 2 mins, before a wash and commencement of imaging at 5 mins. Over the subsequent 90 mins, apelin647 membrane fluorescence, co-localising with the WGA-555 membrane marker, decreased with a corresponding clustering of intracellular fluorescence (**Figure 4A**). At 30 mins, substantial cytosolic fluorescence was already visible. By the end point, distinct intracellular fluorescence was observed where apelin647, presumed bound to internalised apelin receptor, localised to perinuclear compartments with signal occurring adjacent to cell nuclei. **Figure 4B** shows the quantification of apelin647 internalisation, where membrane fluorescence at the start of imaging (normalised to 100 ± 1.94% at 5 mins) reduced by over half (39.30 ± 0.76%) at 90 mins. In contrast, intracellular apelin647 fluorescence increased over the time-course, from 1.94 ± 1.07% at 5 mins to 28.99 ± 0.29% at 90 mins, as the ligand moved from the membrane to the intracellular compartment.

### Apelin647 identifies endogenous apelin receptor in clinically relevant stem cell-derived cardiomyocytes

**Figure 5:**
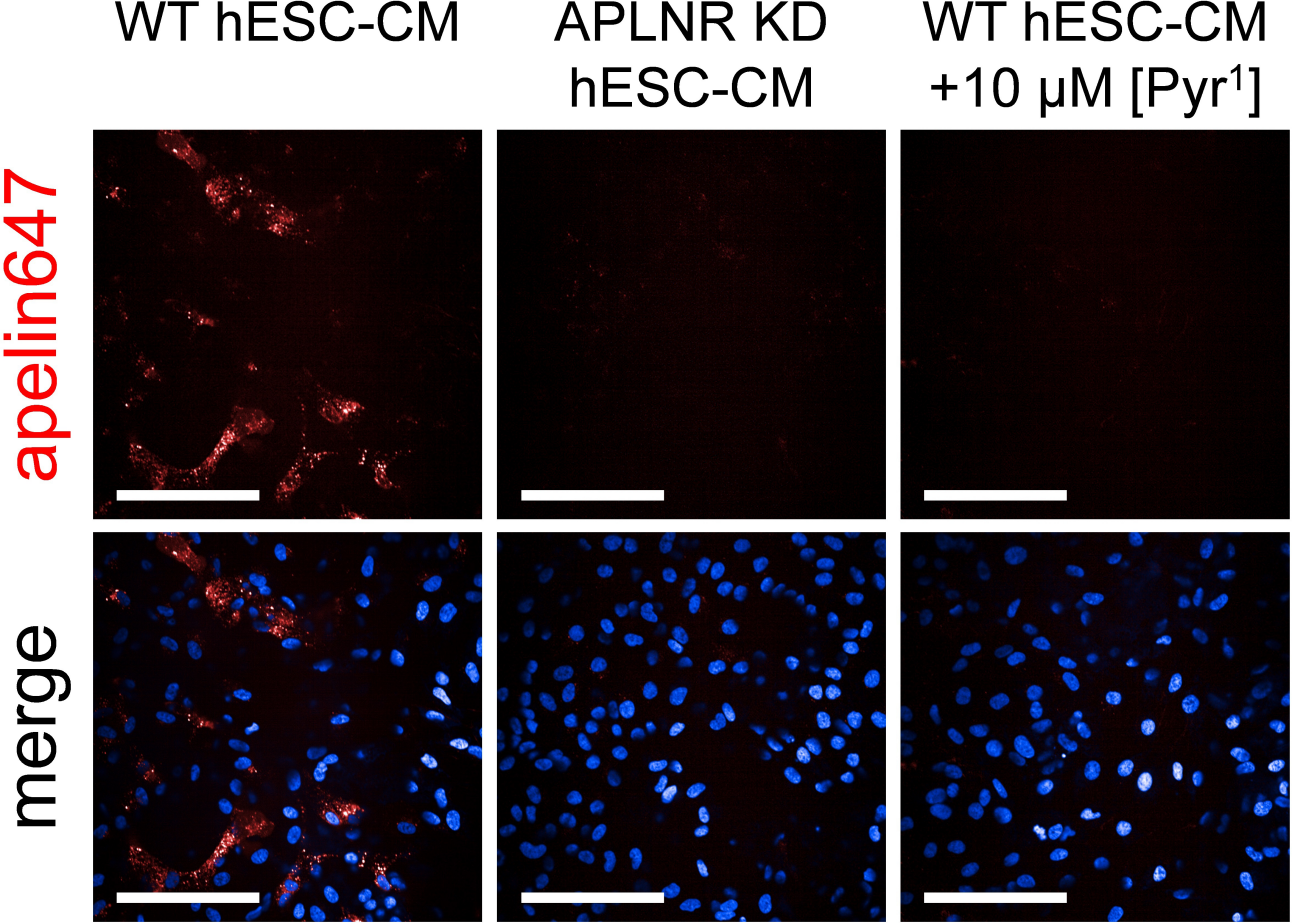
Representative confocal fluorescent images of human embryonic stem cell-derived cardiomyocytes; either wild-type (WT hESC-CM) or apelin receptor knockdown (APLNR KD hESC-CM), treated with 300 nM apelin647 (visualised in red). Non-specific binding was determined in WT hESC-CM cells treated with 300 nM apelin647, in the presence of a saturating 10 µM concentration of unlabelled [Pyr^1^]apelin-13. Bottom row shows a merge with Hoechst 33342 nuclear marker (visualised in blue). n = 4 independent experiments using 4 distinct hESC-CM differentiations. Scale bars indicate 100 µm.

Imaging was used to confirm the suitability of apelin647 in fluorescent staining experiments, where binding of a 300 nM concentration of the ligand was observed in clinically relevant hESC-CMs that endogenously express the apelin receptor (**Figure 5**). Further, apelin receptor knockdown (APLNR KD hESC-CMs) verified the specificity of apelin647 in these cells, with little to no signal observed in this cell population. Finally, little to no signal was also observed in wild-type hESC-CMs co-incubated with a 10 μM saturating concentration of unlabelled [Pyr^1^]apelin-13, again confirming specificity of apelin647 in this platform.

### Apelin488 and ELA488 identifies endogenous apelin receptor in human kidney tissue sections

**Figure 6:**
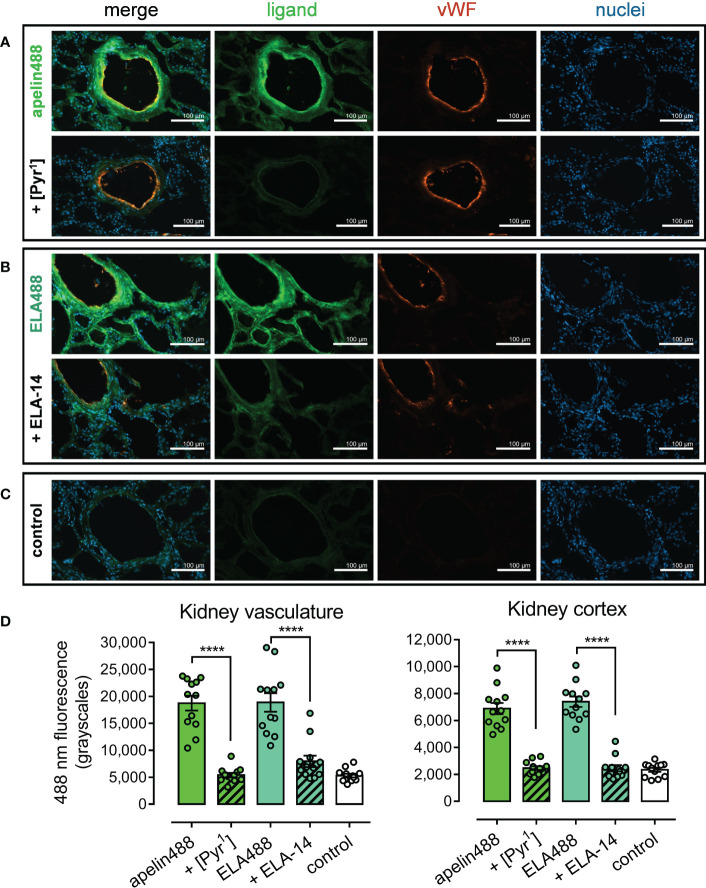
Representative fluorescent images show specific binding of apelin488 and ELA488 to vasculature and cortical tubules in human kidney. **(A)** Kidney sections treated with 1 µM apelin488 (visualised in green), von Willebrand factor (vWF, visualised in orange), and Hoechst 33342 nuclear stain (visualised in blue), in the absence (upper row) or presence (bottom row) of a saturating 10 µM concentration of unlabelled [Pyr^1^]apelin-13. **(B)** Kidney sections treated with 1 µM ELA488 (visualised in green), von Willebrand factor (vWF, visualised in orange), and Hoechst 33342 nuclear stain (visualised in blue), in the absence (upper row) or presence (bottom row) of a saturating 10 µM concentration of unlabelled ELA-14. **(C)** Control panels show kidney sections treated with HBSS buffer and Hoechst 33342 nuclear stain alone (i.e. demonstrate background auto-fluorescence levels). Scale bars show 100 µm in all images. Images representative of tissue from 3 independent donors. **(D)** Bar charts show quantitative 488 nm fluorescence (grayscales) for kidney vasculature structures (left) or tubules in the cortex (right) treated with: 300 nM apelin488 alone or in the presence of 10 µM unlabelled [Pyr^1^]apelin-13; 1 µM ELA488 alone or in the presence of 10 µM unlabelled ELA-14; or with HBSS buffer (control). Data are expressed as mean ± s.e.m., n = 12 individual data points pooled from 3 independent donors. Statistical significance was determined using a one-way ANOVA, with Tukey’s correction for multiple comparisons looking for differences between treatment groups. ****p < 0.0001.

Apelin488 (1 µM) and ELA488 (1 μM) fluorescent ligands were used to identify apelin receptor expression in human kidney tissue sections (**Figure 6**). Apelin488 bound to apelin receptor in tubules of the renal cortex, with particularly intense fluorescence observed in the endothelia of vascular structures, identified with an anti-von Willebrand factor antibody (**Figure 6A**). ELA488 was also observed binding to apelin receptor in tubules and in the endothelium (**Figure 6B**). Fluorescent signal for both apelin488 and ELA488 was attenuated by co-incubation with saturating 10 μM concentrations of the respective unlabelled [Pyr^1^]apelin-13 and ELA-14 parent ligands. Control kidney sections (**Figure 6C**) were treated with HBSS buffer and secondary antibody alone, indicating background fluorescence levels for the channels used to visualise the fluorescent ligands and von Willebrand factor antibody.

Imaging was quantified in kidney sections (**Figure 6D**). In renal blood vessels, mean apelin488 fluorescence (18745 ± 4755 grayscales) was significantly reduced in the presence of [Pyr^1^]apelin-13 (5409 ± 1408 grayscales), where fluorescence did not differ significantly from control fluorescence (5308 ± 1177). Mean ELA488 fluorescence (18869 ± 5968 grayscales) was significantly reduced in the presence of ELA-14 (7946 ± 3656 grayscales), which also did not differ significantly from control fluorescence.

Levels of fluorescence were lower overall in tubular structures of the renal cortex. Mean apelin488 fluorescence (6882 ± 1465 grayscales) was again significantly attenuated by co-incubation with [Pyr^1^]apelin-13 (2461 ± 486 grayscales), whilst mean ELA488 fluorescence (7392 ± 1338 grayscales) was significantly attenuated by co-incubation with ELA-14 (2458 ± 801 grayscales). Neither [Pyr^1^]apelin-13 or ELA-14 co-incubated tubular structures exhibited fluorescence significantly higher than control treated sections (2333 ± 519 grayscales), suggesting that inclusion of unlabelled ligands reduced fluorescence to background levels in all instances.

## Discussion

The apelin receptor is a promising therapeutic target in cardiovascular diseases such as PAH, but is yet to be fully exploited (36, 52). Expanding the apelin receptor toolbox should help design and identify novel agents that will display the necessary affinity, efficacy, and pharmacokinetics to assess the clinical potential of drug-like molecules that bind to the apelin receptor. Novel fluorescent ligands may further delineate differences in receptor binding kinetics, spatio-temporal interactions, and downstream signalling of the two structurally distinct peptide ligands, apelin and ELA, in order to better understand their significance in this unique Class A GPCR-two ligand family. We report the design and validation of four novel fluorescent ligands, two based on the endogenous ligand [Pyr^1^]apelin-13, and two on the second endogenous ligand, ELA.

The structures of the fluorescent peptides are provided in **Figure 1**, alongside the unlabelled endogenous peptides. The incorporation of the non-natural amino acid norleucine at position 11 and tyrosine-O-benzene at position 13 have been shown previously to increase affinity (45), and are incorporated in the iodinated apelin-13 analogue, [^125^I]-apelin-13. The PEG4 linker was chosen to conjugate the fluorophore to the peptides following the success of this strategy in linking anti-serum albumin domain antibodies to apelin peptides (53). Apelin and ELA peptides bind to the receptor with their C-terminus inserted down into the binding pocket (54, 55). Accordingly, we selected to label our fluorescent ligands at the N-terminus, with the rationale that the PEG4 linker and fluorophore would extend out of the binding site, reducing steric hindrance and allowing the peptide to access the pocket. This is an important consideration when labelling a ligand (4–10). The fluorophores themselves (structurally identical to Alexa Fluor 488 and 647 dyes) were selected for their reported high photostability, several fold higher brightness compared to other dyes such as lissamine rhodamine or Texas Red (56), and, as they have recently come out of patent, they are available at much cheaper costs.

Experiments validating our fluorescent ligands (**Figure 2**) showed that, whilst the incorporation of the fluorophores *via* a PEG4 linker did typically reduce the affinity of the ligands for the receptor, they still bound specifically (**Figure 2A**). Reduced affinity may be a consequence of steric hindrance induced by the fluorophore and/or linker. Fluorescent ELA ligands typically showed lower affinity than fluorescent apelin ligands but this might reflect the improved affinity of apelin488 and apelin647 following the inclusion of the non-natural amino acids, or the fact that unlabelled ELA-14 displayed a lower affinity than [Pyr^1^]apelin-13 in our hands. Regardless, the fluorescent ligands behaved as agonists in a functional assay (**Figure 2B**), indicating that the fluorophore or linker did not significantly change the pharmacological profile of the ligands. The fluorescent ligands also triggered receptor internalisation (**Figure 2C**), with apelin647 exhibiting an EC_50_ comparable to unlabelled [Pyr^1^]apelin-13. Apelin647 also displayed the highest affinity of the fluorescent ligands, and we considered this ligand most suitable for subsequent *in vitro* studies. Far-red fluorophores also have the advantage of reduced interference with autofluorescence emitted by endogenous proteins and spectral overlap with commonly used fluorescent proteins such as GFP (4).

Apelin647 binding in CHO-APLNR (**Figure 3**) was concentration-dependent (**Figure 3A**), highly specific (**Figures 3B, C**) and saturable (**Figure 3D**), indicating that the fluorescent ligand was indeed suitable for qualitative and quantitative imaging studies. As proof-of-principle, several apelin receptor ligands were used to screen against apelin647 in CHO-APLNR. The endogenous peptides ([Pyr^1^]apelin-13 and ELA-14), the reported peptide antagonists (MM54 and F13A), and a G protein-biased small molecule agonist (CMF-019), were all able to compete for binding in a concentration-dependent manner (**Figure 3E**). Live cell high content imaging has been used previously in conjunction with a fluorescent adenosine A_3_ receptor antagonist to screen for novel hits (57), and our data suggest that a similar approach could be applied to the apelin receptor. By qualitatively and quantitatively assessing fluorescent apelin receptor signal in wells treated with a library of compounds, hits that bind and compete, and thus reduce fluorescence, could be rapidly and robustly identified.

Apelin receptor fluorescent ligands have been used to track apelin receptor internalisation (42–44). However, studies have typically been limited to exposure of cells to apelin ligand for a specified time and capturing a single snapshot image of the internalised, ligand-bound receptor in a small sample of cells. The brightness and photostability of our selected fluorescent dyes has now allowed visualisation of apelin receptor internalisation over a 90 mins time-course, imaging every 30 seconds in a population of live cells (**Figure 4**). Clear internalisation was observed over time, with movement of apelin647 fluorescence moving from the membrane to the intracellular compartment, with particularly intense fluorescence observed in peri-nuclear regions by the endpoint. As might be expected, intracellular apelin647 fluorescence remained co-localised with wheat-germ agglutinin-555, likely indicating endosomal-dependent internalisation of ligand-bound receptor complexes. Quantification of apelin647 fluorescence over the 90 mins time-course, confirmed that loss of membrane fluorescence over time corresponded with a reciprocal increase in intracellular fluorescence (**Figure 4B**). It would be of great interest to compare the internalisation kinetics of ELA fluorescent peptide to apelin647 in subsequent studies.

We anticipate that future studies using this platform and endosomal/lysosomal specific immunological markers would fully elucidate the mechanisms by which the apelin receptor is internalised. Future studies should examine whether there are differences in the mechanisms and kinetics of internalisation between apelin and ELA-based fluorescent ligands, or assess discrepancies in internalisation between different cell types. Fully understanding internalisation will be crucial in therapeutically targeting the apelin receptor, where avoiding desensitisation and internalisation pathways is hypothesised to be beneficial in treating associated diseases (36, 51). In PAH for example, it has been suggested that G protein-biased agonists, which avoid β-arrestin dependent internalisation, would help maintain the apelin receptor target at the cell surface and prolong increases in vasodilatation and cardiac output induced by therapeutic agents (36, 51).

Another advantage fluorescent ligands offer is their potential for use as probes to determine high resolution expression and distribution of protein targets in cells and tissue (4). We have used a similar strategy to show binding of a fluorescent SARS-CoV-2 viral spike protein monomer to the host target protein, angiotensin-converting enzyme 2, in human kidney, heart, lung, and liver tissue (58). Apelin647 bound to apelin receptor endogenously expressed in a clinically relevant hESC-CM cell model (**Figure 5**). An inducible knockdown strategy (48, 49) was used to generate an apelin receptor null hESC-CM line, which did not bind apelin647 and confirmed the specificity of the fluorescent ligand. Detectable signal for apelin647 binding to native receptors in a cell model suggests that the fluorescent ligand could be used as a suitably sensitive replacement to visualise apelin receptor protein in imaging and flow cytometric platforms where antibodies may be unavailable or unreliable.

Fluorescent ligands have been used previously to characterise the expression of GPCRs in tissue samples. As an example, the synthesis of several derivatives of the CAY10583 ligand, conjugated to fluorescein, resulted in the development of a fluorescent ligand targeting the leukotriene B4 receptor 2 (BLT2) GPCR (59). The ligand was shown to bind specifically to the BLT2 target protein, and was used to visualise expression of BLT2 in murine skin explant, where fluorescence was completely abolished by co-incubation with unlabelled ligand. Owing to technical difficulties with our chosen imaging platform, apelin647 could not be visualised in tissue samples. Instead, however, apelin488 and ELA488 fluorescent ligands were used to examine the expression and distribution of apelin receptor in human renal tissue (**Figure 6**). As expected, both apelin488 (**Figure 6A**) and ELA488 (**Figure 6B**) bound specifically to apelin receptor present in blood vessels in the kidney, where it co-localised with an anti-von Willebrand endothelial cell marker (26–30, 34). Additionally, lower levels of apelin488 and ELA488 fluorescence were observed in the tubules of the renal cortex. This is the first time, to our knowledge, that a fluorescent ligand has been used in this way to explore endogenous apelin receptor in human tissue. The pattern of distribution closely matched previous findings in an immunohistological study assessing apelin expression in the human kidney (60). One limitation of using 488 nm wavelength fluorophores in tissue section imaging is that there can be considerable interference by autofluorescence of natural proteins such as elastin. However, negative control sections treated with buffer alone (**Figure 6C**), show that apelin488 and ELA488 are sensitive and bright enough to be visualised above background fluorescence in these samples (quantification shown in **Figure 6D**). These data suggest that both apelin- and ELA-based fluorescent ligands could be employed as safer and easier-to-use replacements for radiolabelled ligands in autoradiographical techniques. Additionally, fluorescent ligands may provide considerably better spatial resolution of the expression and distribution of the apelin receptor, whilst also affording the inclusion of other fluorescent markers to identify specific tissue structures and cell types that express the protein. Future studies will also aim to confirm the suitability of apelin647and ELA647 in renal tissue sections.

## Conclusions

We outline the design and validation of four novel fluorescent apelin receptor ligands; two based on apelin (apelin488 and apelin647), and two based on ELA (ELA488 and ELA647). The fluorescent ligands were able to bind to the receptor and retained agonist pharmacological profiles. We used these ligands to characterise the apelin receptor at multiple levels, ranging from subcellular binding, expression, and internalisation, through to tissue distribution in human renal samples. We also demonstrate proof-of-principle that these fluorescent ligands could be used to screen for hits against the apelin receptor in drug discovery pipelines to identify new compounds that may have clinical use in cardiovascular diseases such as PAH. Next steps should aim to use these ligands to elucidate differences in the pharmacological and kinetic profiles of apelin and ELA peptides.

## Data availability statement

The raw data supporting the conclusions of this article will be made available by the authors, without undue reservation.

## Author contributions

TW, RM, and RK designed and carried out experiments and data analysis. AB contributed grant support and facilities. JM and AD designed and supervised experiments and data analysis, and contributed grant support and facilities. All authors contributed to the writing and/or review of the manuscript. All authors contributed to the article and approved the submitted version.
